# A systematic review of tools used to screen and assess for externalising behaviour symptoms in low and middle income settings

**DOI:** 10.1017/gmh.2019.11

**Published:** 2019-07-15

**Authors:** B. Nezafat Maldonado, J. Chandna, M. Gladstone

**Affiliations:** Department of Women and Children's Health, Institute of Translational Medicine, University of Liverpool, Alder Hey Children's NHS Foundation Trust, Eaton Road, Liverpool, L12 2AP, UK

**Keywords:** Assessment, behaviour, child, cross-cultural, low and middle income, measurement, screening, review

## Abstract

**Background.:**

Mental health issues, often manifested as behavioural difficulties, in children are estimated to be high in low and middle-income countries (LMIC) settings. There is a paucity of definitive data due to a lack of well-validated tools to use across settings. This review aims to provide evidence on what tools are used and which have been adapted and validated in LMIC settings.

**Methods.:**

We performed a systematic review to identify tools used to assess or screen externalising behaviour problems in children and adolescents in LMIC and assess their cultural adaptations. We searched for studies measuring externalising behaviour in children from 0 to 19 years published up to September 2018. Articles were assessed to identify tools used and analysed using the Ecological Validity Framework.

**Results.:**

We identified 82 articles from over 50 LMICs who had studied externalising behaviour in children. Twenty-seven tools were identified, with a predominance of studies using tools from the USA and Europe. Most studies did not describe an adaptation and evaluation process, with only one study following recommended criteria. New tools were identified which both screen and assess externalising behaviour which have not yet been utilised across settings.

**Conclusions.:**

Although tools from the USA and Europe are often utilised to screen and assess for externalising behaviour problems in children in LMICs, the conceptual frameworks behind the use of these tools in other cultural contexts are not always carefully examined. In order to have valid data across cultures, we should aim to adapt and validate tools before use. Provision of processes to validate tools across LMIC settings would be beneficial.

## Introduction

Mental health difficulties account for over 20% of the global burden of years lived with disability in low and middle-income countries (LMIC) (Vos *et al*. 2012; Becker & Kleinman, 2013; Mokdad *et al*. 2018). In many of these countries, over 50% of their population are under the age of 14 years (The World Bank, 2016) with a high percentage of children at risk of mental health and behavioural difficulties (Collins *et al*. 2011; Kieling *et al*. 2011). Exposures that affect brain development and function are endemic in many of these settings and are likely to cause the high rates of behavioural difficulties seen (Rodríguez-Barranco *et al*. 2013). This includes exposures to infections such as cerebral malaria, meningitis, encephalitis and HIV, perinatal problems and premature birth. The impact that these conditions can have on the functional abilities of children is often under-recognised, identified late and can go untreated. Furthermore, a substantial proportion of mental health difficulties (globally) in adults originate early in life, particularly externalising behaviour problems (Merikangas *et al*. 2009). These children often have difficulties with both cognition and self-control, which can manifest as disruptive behaviour (DB) in the form of aggression, rule-breaking, hyperactivity or inattention.

Although the epidemiology clearly demonstrates these high rates of mental health and behavioural problems in children, limited services provide support for them (WHO, 2013). The Mental Health Atlas 2014 shows almost a complete lack of data for the diagnosis and treatment for child mental health conditions in LMIC (WHO, 2014). Despite this, the WHO Mental Health Action Plan 2013–2020 highlights the dire need for better mental health support in low-income settings (WHO, 2013). The Mental Health Gap Action Programme (mhGAP); launched by the WHO in 2016 has tried to address this with some pragmatic approaches to child mental health. This has brought an enhanced commitment by some countries to improve the treatment and assessment of mental health and psychiatric conditions; including those for children (WHO, 2008).

In order to implement programmes around child's mental health and development, we need to be able to identify children with mental health disorders more appropriately in a variety of different cultural settings. Much of the paucity of specific data on mental health and behavioural problems in children in LMIC settings relates to the lack of tools, which can identify and assess behaviour in these settings. The use of a wide variety of often, not well-validated tools, can also lead to a lack of compatibility between studies. In recent years, the global mental health community has tried to promote research on ensuring that tools for assessing mental health and neurodevelopment in children are validated for the particular cultures and settings they are used in (Collins *et al*. 2011; Kieling *et al*. 2011). There are presently no guides provided by this wider mental health community as to which tools to use for this purpose.

Mental health issues in children can be classified as internalising or externalising, depending on the symptoms that are presented (Achenbach & Edelbrock, 1978). We can define externalising behaviour problems or disorders where behavioural symptoms cause the child to act negatively on the external environment, i.e. symptoms seen by those around patients. This group of behavioural problems includes but it is not limited to, uncontrolled aggressive conduct disorders, disruptive behaviour, attention deficit hyperactivity disorder (ADHD) (Huesmann *et al*. 1987).

In contrast, children may develop internalising behavioural problems that affect the child's internal psychological environment rather than the external surroundings. These problems include anxiety and depressive symptoms. The distinction between the two categories is not perfect and the two overlap. A child's internalising behavioural problems can have a negative impact on other people around them and a child's externalising behaviour problems can have internal psychological implications. The distinction is useful clinically in considering treatments for children. Research studies have also demonstrated the longitudinal nature of these conditions with children who have conduct disorders more likely to grow up to be violent as adults and children with internalising behavioural problems more likely to develop depression in the future (Fryers & Brugha, 2013). Clinically, it is vital that children who are being assessed for behavioural difficulties should be assessed for both externalising and internalising problems. Furthermore, mental health assessments should aim to explore dimensional psychological constructs that are relevant to human behaviour and mental disorders. The Research Domain Criteria (RDoC) recommended the use of multiple methodologies for assessing children and that we also take into account developmental trajectories and environmental influences alongside our assessment (Insel *et al*. 2010).

In high-income countries, mental health practitioners will often use tools to provide information on the presence and severity of behaviours. These tools are varied and can include questionnaires or checklists that provide information on the internalising and externalising characteristics or behaviours of an individual child. Mental health practitioners may ask parent or teachers to report on behaviours through the use of these tools and in some cases, they are observational. Often practitioners use them to provide information to enable diagnostic labels to be given to some children. Common examples include The Achenbach Child Behaviour Checklist (CBCL) (Achenbach, 1991, 2009), the Conners' Rating Scales (Conners *et al*. 1998), the Behaviour Assessment System for Children (BASC) (Reynolds & Kamphaus, 2015). Widely used screening tools include the Strengths and Difficulties Questionnaire (SDQ) (Goodman, 1997), the Survey of Well-Being of Young Children (SWYC) (Sheldrick *et al*. 2012) or the Ages and Stages Socio-emotional screener (Squires *et al.* 1997). This behavioural screening tool for 3–16 year olds includes 25 items that aims to screen for emotional symptoms, conduct problems, hyperactivity, peer relationships and prosocial behaviour (Goodman, 1997; M. *et al*. 2008). The SDQ has now been translated and validated for over 40 countries. Many of these are, although not exclusively, from high-income settings (Stone *et al*. 2010). Despite the widespread use of these measures for screening and assessment of behavioural problems, many of these tools reflect a psychological literature that is derived largely from Western industrialised societies (Henrich *et al*. 2010). Presently, there is little information on the variety and robustness of tools created or adapted to screen or support assessment of externalising behaviour problems in LMIC.

Previous research on externalising behaviour problems have associated high prevalence of these mental health problems with academic under achievement and unemployment in a population (Taylor *et al*. 1996; Fergusson & Horwood, 1998; Fergusson & Woodward, 2002). In the context of LMIC, this is of high importance as the negative cycle of poverty and mental ill health is well established, and children, particularly those with externalising behaviour problems are likely to be a substantial economic and social burden. Externalising behaviour problems respond well to early intervention, but further epidemiological data is essential to inform policy and encourage future actions. To study the prevalence of these problems in LMIC, culturally appropriate and accurate tools are necessary.

Experts recommend that practitioners or researchers should ensure adequate cultural adaptation at linguistic and conceptual levels to ensure accurate screening and assessment (Guillemin *et al*. 1993). Developing a new tool for a particular setting can require a high level of expertise and can be resource intensive. Many researchers, therefore, resort to adapting existing well-known tools for their studies. This can be problematic and may lead to bias within studies. Table 1 summarises possible scenarios when some form of cross-cultural adaptations may be required within research studies in LMIC settings.

**Table 1.** Possible scenarios that may require cross-cultural adaptations (adapted from Guillemin *et al*. 1993)

Using a questionnaire in a new population describes as:		Results in a change in…			Requires…	
		Culture	Language	Country of use	Translation	Cultural Adaptation
A	Use in same population. No change in culture, language or country from source	**−**	**−**	**−**	**−**	**−**
B	Use in established immigrants in source country	+	−	−	−	+
C	Use in other country, same language	+	−	+	−	+
D	Use in new immigrants, not speaking of local language but in the same source country	+	+	−	+	+
E	Use in another country and another language	+	+	+	+	+

There are few studies or reviews which provide good information on the variety of tools used for assessing externalising behavioural problems in LMIC settings and none which provide good information on the procedures undertaken to validate tools used within these settings. Without this information, it is difficult for researchers to know what is out there, available and of good quality for use. This structured systematic review aims to address this gap and to search the current literature to identify tools which assess or screen for externalising behaviour problems in children and young people under the age of 19 in LMIC settings. We are particularly focussing on externalising behavioural problems as they can be particularly problematic for families and communities in low and middle-income settings. Furthermore, we felt that focussing on one specific area of behavioural difficulties in children would be most helpful for the field. Our secondary objective is to understand how these tools have been adapted and validated, if at all, to fit with the cultural settings of populations other than those in Western industrialised settings.

## Methodology

We report this review according to the PRISMA guidelines for reporting systematic review (Liberati *et al*. 2009).

We defined *externalising behaviour* or *externalising behaviour problems* as problems or disorders that overall reflect the child negatively acting on the external environment, i.e. symptoms seen by those around patients.

### Search strategy and selection criteria

We included all studies that reported on the use of a measurement tool to identify children and adolescents under the age of 19 years with externalising behaviour problems. We included studies that used tools that were more general and identified children with a range of mental health problems if they also included identifying children with externalising behaviour problems. We discussed any articles where researchers did not clearly define externalising behaviour, prior to making a decision as to whether to keep the article in. We included all articles published in either English, Spanish or Portuguese between 1990 to September 2018. There were no limitations on study design or publication type imposed to ensure an adequate number of studies identified. To be more specific in our search for studies that were identifying children with externalising behaviour problems, we excluded studies that exclusively explored internalising behavioural such as depression or post-traumatic stress disorder. Studies that looked at externalising behaviour using teacher, parent-report or self-reporting were included in the review.

We kept our search broad, to begin with and searched MEDLINE, SCOPUS, Web of Science, and the Cochrane Library and included conference proceedings. We searched using the following terms; ‘externalising behaviour’ AND ‘behaviour problem’ AND ‘child’ AND ‘developing country’ OR ‘low and middle income country’ OR the name of each LMIC, as defined by World Bank 2016 (online Supplementary File 1).

**Fig. 1.**
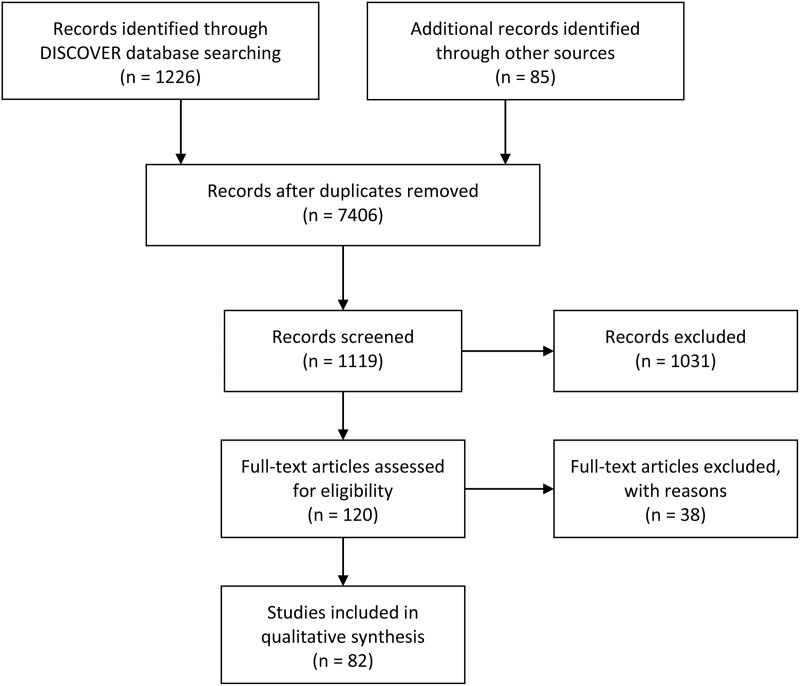
PRISMA diagram of selection of studies in LMIC settings where tools assessing or screening for externalising behaviour problems in children were identified.

We discussed the proposed search strategy with different experts working on this topic prior to starting the search. To identify any unpublished or ongoing studies, we contacted individual researchers working in the field. We reviewed reference lists from all included studies and articles were included when appropriate. The PRISMA flow diagram is shown in Fig. 1.

#### Selection of studies

We imported search results into EPPI-Reviewer software to remove duplicates and screen by title and abstract. Title and abstract screening was performed by two independent reviewers (MG and BNM), with consensus decision in cases of disagreement. We then retrieved full texts and re-assessed against inclusion criteria. Two reviewers scrutinised the full text of all studies, which passed title and abstract. Any disagreements about final inclusion were resolved by a third reviewer (JC). We scrutinised publications for duplicate data. We list studies excluded after full-text assessment and their reason for exclusion in online Supplementary File 2.

## Extracted data

We developed a study characteristic form to extract the relevant data from selected studies and gathered general information on; author(s), country of setting, their purpose, participant's age group, tool used and whether researchers had adapted or validated the tool in any way. We included any studies that also aimed to validate a tool in a new setting. The section on validity in the data collection form was adapted from the Ecological Validity Framework (EVF) model (Bernal *et al*. 1995). The model aims to identify the critical elements in cultural adaptation and has been previously used to assess tools used to screen for autism (Maskari *et al*. 2018). The EVF model has eight components: language, metaphors, person, contents, concepts, methods, goals and context. To facilitate data extraction and standardise the process, questions were developed for each domain with a Yes (+) or No (−) answer (Table 2)

**Table 2.** Ecological Validity Framework (adapted from Bernal *et al*. 1995)

Dimensions	Culturally sensitive elements
Language	Did authors report that tools were translated through forward and back translation process? Were measures put in place to ensure culturally appropriate language was used?
Persons	Ethnic/racial similarities and differences between therapist and patient group was considered and shaped the development of the tool?
Metaphors	Were verbal and/or visual symbols, sayings and concepts common with the population group used to assess externalising behaviour?
Content	Was cultural knowledge on values, costumes and traditions of the population group taken into account when developing the tool?
Concepts	Are efforts to adopt appropriate socio-cultural concepts presented in the study?
Goals	Do the goals of behavioural assessment consider the cultural uniqueness of the target population?
Methods	Were measures in place to facilitate the delivery of the tool within the population's cultural context?
Context	Do authors consider the social, economic and political contexts of behaviours in this population?

## Results

### Number of articles found

The database search yielded 11 226 citations (Fig. 1). In total 3206 studies were duplicates and were removed leaving 7406 paper titles and abstracts to be scrutinised. A total of 346 abstracts were selected after this process. In total 338 were full-text articles retrieved for eligibility assessment against the inclusion criteria; full text was not available for eight papers. Overall, 82 articles were included as part of this review (Table 3).

**Table 3.** Table demonstrating tools to assess and screen for externalising behaviour problems identified in low and middle-income countries

Author	Setting	Purpose of the study	Patient age range	Externalising behavioural outcome measured	Tools used to assess	Adaptation process	Validation process
Chen *et al.* (2015)	China	Prevalence study	6–18 years	Child behaviour	CBCL	Nil	Validation study Leung *et al.*, (2006)
Alarcon Parco and Barrig Jo, (2015)	Peru	Prevalence study	12–18 years	Child behaviour	Youth Self Report	Nil	Nil
Sabet *et al.* (2009)	South Africa	Prevalence Study	12 years	Behavioural outcomes	Youth Self Report	Minor language edits and trained fieldwork used to overcome potential language problems	Nil
Rodriguez Puentes and Cortes Arboleda (2017)	Colombia	Prevalence study	11–19 years	Child behaviour	Youth Self Report Spanish version	Nil	Validated in Spain
Lachman (2011)	Algeria	Prevalence study	6–18 years	Child behaviour	CBCL	French version CBCL	Validated in France
Abdul Kadir *et al.* (2015)	Malaysia	Prevalence study	14–21 years	Child behaviour	SDQ	Translated into Malay	Nil
Abou-Khadra *et al.* (2013)	Egypt	Prevalence study	6–12 years	Child behaviour	CBCL	Arabic version	Nil
Al-Awad & Sonuga-Barke (2002)	Sudan	Prevalence study	6–19 years	Child behaviour	CTRS-39 and CPRS-48 were	Translated and back-translated. Piloted Review by expert panel.	Study was the validation study.
Alckmin-Carvalho *et al.* (2017)	Brazil	Prevalence study	12–14 years	Child behaviour	Youth Self Report	Nil	Validation study Rocha (2012)
Alizzy *et al.* (2017)	Yemen	Prevalence study	11–16 years	Externalising behaviour	SDQ	Nil	Arabic version Almaqrami & Shuwail (2004)
Anselmi *et al.* (2008)	Brazil	Prevalence study	12 years	Child behaviour	CBCL	Nil	Nil
Ashenafi *et al.* (2001)	Ethiopia	Prevalence study	5–14 years	ADHD, Disruptive behaviour, mood and anxiety	Diagnostic Instrument for Children and Adolescents	Translation into Amharic	Validation in separate study in 2000. Full text for validation study not available.
Avan *et al.* (2010)	South Africa	Prevalence study	2 years old	Child behaviour problems	Richman child behaviour scale	‘*Some changes were made to adapt it to the local culture’.*	Nil
Bakare *et al.* (2011)	Nigeria	Prevalence study	15–18 years old	General function in adolescents with bipolar disorder	Child Global Assessment Scale (C-GAS)		Earlier pilot survey done
Baker-Henningham *et al.* (2012)	Jamaica	Prevalence study	3–6 years old	Effects of intervention on conduct problems and social skills	Sutter-Eyberg Student Behavior Inventory (SESBI) – Teacher report Connor's Global Index – Child report Eyberg Child Behavior Inventory (ECBI) – parent report SDQ	Nil	Nil
Bakhshayesh *et al.* (2011)	Iran	Prevalence study	6–14 years old	Effects of intervention on ADHD	German ADHD Rating Scale (DISYPS- KJ)	Nil	Nil
Bangirana *et al.* (2009)	Uganda	Prevalence study	7–16 years	Child behaviour	CBCL	Luganda version	Study is the validation study.
Bangirana *et al.* (2011)	Uganda	Prevalence study	5–12 years old	Cognition, behaviour and academic skills in children surviving severe malaria	Kaufman Assessment Battery for Children (KABC-II) CBCL	KABC II- adapted	KABC II – validated for Uganda CBCL – validated Luganda version
Betancourt *et al.* (2010)	Sierra Leone	Prevalence study	10–17 years	Child behaviour	Oxford Measure of Psychosocial Adjustment	Developed and validated for use in Sierra Leone	Study is validation study.
Betancourt *et al.* (2012)	Ethiopia	Prevalence study	11–18 years	Child behaviour	CBCL Kunamenga adaptation of the Youth Self Report	Nil	Previously used Mulatu (1995)
Bilici *et al.* (2004)	Turkey	Prevalence study	6–14 years	Effects of intervention on ADHD	− DuPaul Parents Rating ADHD – Turkey version ADHD Scale developed by author Turkish Conners (TACTQ)	All Western tools were adapted, or new tool was developed	Du Paul previously validated for Turkey TACTQ (Conners, 1990) was used in this study (Dereboy *et al.* 1997).
Burlaka (2016)	Ukraine	Prevalence study	9–16 years	Externalising behaviour	CBCL	Translated into Ukrainian, back-translated and reviewed by a panel. Pilot	Nil
Cao *et al.* (2013)	China	Prevalence study	6–11years	Child behaviour	Conners Parent Symptom Questionnaire	Nil	Nil
Sharp *et al.* (2014)	South Africa	Prevalence study	7–11 years	Child behaviour	SDQ Computerized Diagnostic Interview Schedule for Children – 4th Edition (CDISC-IV)		CDISC-IV was validated for use with Sesotho families
Dagar *et al.* (2011)	India	Prevalence study	<18 years	Child behaviour	CBCL	Nil	Nil
Dave *et al.* (2014)	India	Prevalence study	6–12 years	Effects of intervention on ADHD	DSM Adapted Questionnaire		Nil
DiGirolamo *et al.* (2010)	Guatemala	Prevalence study	6–11 years	Effect of zinc supplementation on the mental health	Behavioural Assessment System for Children (BASC)		Version was previously validated for use in Colombia
Ding *et al.* (2014)	China	Prevalence study	2–3 years	Behavioural problems	SDQ	Vietnamese version used	Nil
Eloff *et al.* (2014)	South Africa	Prevalence study	6–10 years	Child Behaviour	CBCL	Nil	Nil
Erdogan *et al.* (2008)	Turkey	Prevalence study	5–7 years	Behavioural problems and enuresis	CBCL	Turkish Version	Validated previously. Erol *et al.* (1995)
Familiar *et al.* (2015)	Uganda	Prevalence study	5–12 years		CBCL Behaviour Rating Inventory of Executive Function (BRIEF)	CBCL was previously translated and adapted by Bangirana (2009) in a two-step process. BRIEF was translated into Lugan (forward and back-translated). Final version was approved by authors	Nil
Farahat *et al.* (2014)	Egypt	Prevalence study	Primary school	Child behaviour	Conners Rating Scales	Arabic version	Nil
Farcas *et al.* (2017)	Romania	Prevalence study	5–11 years	Hyperactivity	CBCL SDQ	Hungarian version of SDQ	Nil
Ghanizadeh *et al.* (2013)	Iran	Prevalence study	5–16 years	Effects of intervention on ADHD	Persian version of DSM-IV based parents’ ADHD rating questionnaire	Tool was created for this population	Previously validated. Ghanizadeh & Jafari (2010)
Kariuki *et al.* (2012)	Kenya	Prevalence study	6–9 years	Behavioural problems	Child Behavior Questionnaire for Parents (CBQFP)		Previously used in this population – no measure of validity provided
Kerfoot *et al.* (2007)	Ukraine	Prevalence study	6–17 years	Child behaviour	SDQ	Translated into Russian	Nil
Kippler *et al.* (2012)	Bangladesh	Prevalence study	5 years	Child development	SDQ	Translated and piloted with target population.	Test–retest reliability at 7-day intervals (intraclass correlation *r* > 0.90) measured during pilot
Lachman (2013)	South Africa	Prevalence study	N/A	Child Behaviour	SDQ	Nil	Nil
Lagunju *et al*. (2012)	Nigeria	Prevalence study	7–14 years	Child behaviour	Child Behaviour Questionnaire Rutter Scale A2	Translated to Yoruba and interpreter used to aid participants	Nil
Lambert & Lyubanksy (1999)	Jamaica	Prevalence study	6–16 years	Externalising behaviour	Jamaica Youth Checklist (JYC)	Modelled after the CBCL but sensitive to Jamaican culture	Validation Lambert *et al.* (1994)
Liu *et al.* (2001)	China	Prevalence study	6–16 years	Child behaviour	CBCL	Nil	
Liu *et al.* (2009)	Mauritius	Prevalence study	11 years	Child Behaviour	CBCL	‘Previously used in this population’.	Nil
Louw *et al.* (2016)	South Africa	Prevalence study	10–16 years	Behavioural problems	CBCL	‘Previously used in this population’.	Ni
Meftagh *et al.* (2014)	Iran	Prevalence study	8–10 years	Effects of intervention on ADHD	Child Symptoms Inventory (CSI-4)	Nil	Nil
Menon *et al.* (2007)	Zambia	Prevalence study	11–15 years	Child behaviour	SDQ	Translated and back translated to local dialect	Nil
Mpang *et al.* (2017)	Uganda	Prevalence study	5–18 years	Child behaviour	Child and Adolescent Symptom Inventory (CASI-5)	6 stages to adaptation	Validation study
Munir & McConachie (1999)	Bangladesh	Prevalence study	2–9 years	Child behaviour	Independent Behaviour Assessment Scale (IBAS)		Study is the construction of the tool
O'Callaghan *et al.* (2014)	Democratic Republic Congo	Prevalence study	7–18 years	Internalising and externalising behaviour	African Youth Psychosocial Assessment Instrument (AYPA)	*‘The AYPA was translated, back-translated, submitted to a focus group and piloted before being administered’.*	AYPA was developed in northern Uganda. Betancourt *et al.* (2014)
Omigbodun *et al.* (1996)	Nigeria	Prevalence study	7–14 years	Child behaviour	Children's Behaviour Questionnaire Rutter Scale A2 Reporting Questionnaire for Children (RQC)		Study is validation study
Panter-Brick *et al.* (2009)	Afghanistan	Prevalence study	5–16 years	Child behaviour	SDQ	Dari and Pashto version developed	Nil
Perera *et al.* (2012)	Sri Lanka	Prevalence study	4–12 years	Effects of intervention on ADHD	SNAP-IV checklist	Nil	Nil
Pires *et al.* (2012)	Brazil	Prevalence study	6–13 years	Analysis of ADHD related factors	CBCL	Previously adapted and validated	CBCL Brazilian version was previously validated (Bordin *et al.* [2013])
Prasad *et al.* (2014)	India	Prevalence study	3–18 years	Behaviour Dysfunction of Children with Neurocysticercosis	CBCL	Nil	Nil
Ramchandani *et al.* (2010)	South Africa	Prevalence study	2 and 4 years	Maternal prenatal stress and child behaviour	Richman Behaviour Screening Questions	Nil	Nil
Rimal & Pokharel (2016)	Nepal	Prevalence study	11–17 years	Child behaviour	SDQ	Nil	Nil
Rimal (2013)	Nepal	Prevalence study	11–17 years	Child behaviour	SDQ	Nil	Nil
Rochat *et al.* (2016)	South Africa	Prevalence study	7–11 years	Breastfeeding and child behaviour	CBCL	*‘… which has been validated across multiple cultural settings’*	Nil
Roy *et al.* (2009)	India	Prevalence study	3–7 years	Lead exposure and child behaviour	Conners’ ADHD/Diagnostic and Statistical Manual for Mental DSM-IV Scales (CADS-T) Conners ‘Teachers Rating Scale Behaviour rating Inventory of Execution Fucntion(BRIEF)	All tools were translated into Tamil	Nil
Rus *et al.* (2016)	Romania	Prevalence study	6–18 years	Child behaviour	CBCL	Nil	Nil
Sahu *et al.* (2013)	India	Prevalence study	6–15 years	Child behaviour	Conners 3 Parent Rating Scale (Short)	Translated into Hindi	Nil
Samad *et al.* (2005)	Pakistan	Prevalence study	4–16 years	Child behaviour	SDQ	Translated and back-translated from English to Urdu.	Study was the validation study.
Samarakkody *et al.* (2010)	Sri Lanka	Prevalence study	4–6 years	Child behaviour	Child Behaviour Assessment Instrument (CBAI)	Development of new tool	Statistical measures: Cronbach's *α* to assess validity
Sánchez *et al.* (2011)	Panama	Prevalence study	6–11 year	ADHD prevalence	Behavioral Assessment System for Children (BASC-2) Conner's Parents Rating Scales	Spanish versions	Nil
Sanmaneechai *et al.* (2005)	Thailand	Prevalence study	3–5 years	Child behaviour	CBCL	Nil	Nil
Santos *et al.* (2013)	Brazil	Prevalence study	6–16 years	Effects of intervention on ADHD	CBCL Swanson, Nolan and Pelham Questionnaire (SNAP-IV)	No details provided	CBCL was validated previously for use in Brazil (Bordin *et al.* [2013])
Santos *et al.* (2015)	Brazil	Prevalence study	4 years	Child behaviour	CBCL	*‘Previously adapted and validated’*	CBCL Brazilian version was previously validated (Bordin *et al.* [2013])
Shahini *et al.* (2015)	Kosovo	Prevalence study	6–18 years	Child behaviour	CBCL	Nil	Nil
Sipsma *et al.* (2013)	South Africa	Prevalence study	6–10 years	Child behaviour	CBCL	Translated and back-translated *‘Piloted and reviewed for conceptual applicability and cultural relevance’.*	Nil
Syed *et al.* (2007)	Pakistan	Prevalence study	5–12 years	Child behaviour	SDQ	‘Validated version used’	Nil
Tadesse *et al.* (2012)	Ethiopia	Prevalence study	6–14 years	Child behaviour	CBCL Achenbach System of Empirically Based Assessment	Nil	Nil
Thabet *et al.* (2010)	Palestine	Prevalence study	6–15 years	ADHD prevalence	SDQ	Arabic version, previously validated in Yemen and Gaza strip	Validation study Alyahri *et al.* (2006),
Thabet *et al.* (2000)	Gaza	Prevalence study	3–16 years	Child behaviour	SDQ	Translated, back-translated and reviewed. Pilot	Nil
Ulloa *et al*. (2006)	Mexico	Prevalence study	6–11 years	ADHD symptoms	CBCL	*‘Previously used in this population’*	Nil
Walker *et al.* (2010)	Jamaica	Prevalence study	6 years	Child behaviour	SDQ	*‘Minor modifications on language’*	Nil
Özmert *et al.* (2002)	Turkey	Prevalence study	Pre-school	Child behaviour	CBCL	‘*Previously adapted and validated’*	Nil
Hartini *et al.* (2015)	Indonesia	Validation study	6–18 years	Validation of tool	CBCL	Translated	Nil
Albores-Gallo *et al.* (2016)	Mexico	Validation study	1–5 years	Validation of tool	CBCL	Translated into Spanish – Mexican, then back translated, reviewed and piloted	Cronbach's *α* and Pearson correlation were measured of validation used
Brasil & Bordin (2010)	Brazil	Validation study	6–14 years	Child behaviour	CBCL	Nil	Validation by Bordin (1995)
Dang *et al.* (2017)	Vietnam	Validation study	6–16 years	Increment validity	CBCL SDQ	CBCL was translated and back-translated SDQ Vietnamese version was used	Validation study for CBCL SDQ previously used
Eslami *et al.* (2010)	Iran	Validation study	15–19 years	Externalising behaviour	Adolescent Health and Development Questionnaire	Translation and back translation followed by pilot study	Validation study
Kariuki *et al.* (2016)	Kenya	Validation study	6–9 years	Evaluation of psychometric properties	CBCL	Translated to Ki-Swahilli	Cronbach's *α* = 0.95 and ITC Test-retest reliability (r = 0.76; *p* < 0.001).
Mukherjee *et al.* (2014)	India	Validation study	6–9 years	Evaluation of Psychometric properties	INDT-ADHD and CPRS	Developed by an expert panel	Validation study

SDQ, Strengths and Difficulties Questionnaire; CBCL, Child Behaviour Checklist; CTRS, Conners' Teacher Rating Scale; CPRS, Conners’ Parent Rating Scale.

### Countries of origin

We found over 50 LMICs that were measuring externalising behaviour using a questionnaire-based approach (Table 5). This included settings in Africa (Sudan, South Africa, Kenya, Nigeria, Democratic Republic Congo, Uganda, Zambia, Sierra Leone and Ethiopia), Asia (Indonesia, China, Malaysia, India, Sri Lanka, Bangladesh, Nepal, Vietnam, Afghanistan), Middle East (Iran, Turkey, Palestine, Egypt), Central and South America (Brazil, Jamaica, Panama, Mexico) and Europe (Ukraine and Romania). In addition, areas that have been recently subjected to conflict and humanitarian emergencies such as the Gaza strip (Thabet *et al*. 2000) and Kosovo (Shahini *et al*. 2015) were also using tools in studies. Most published research on externalising child behaviour in LMICs originated from the African region, compared to Asia, Middle East and South and Central America.

### Types of study

Most studies included in the review were cross-sectional studies providing a one-point snapshot of the prevalence of externalising behaviour. The tools used were variable and conclusions about children having externalsing behaviour symptoms or features of ADHD sometimes came only from the use of screenng tools such as the SDQ. This included a study in Nepal where the SDQ was used to identify children with behavioural difficulties in order to measure prevalence rates of ADHD among school children. The studies we found were quite variable with data collected in some cases, only from the child, in other studies, only from the parent and in other studies, only from teachers. This included a study in Zambia, which examined prevalence of child behavioural problems in HIV-positive adolescents by only using the youth- report version of the SDQ (Menon *et al*. 2007). Some studies, such as that in Palestine, used both parent and teacher-reported Arabic SDQ scores to establish the prevalence of externalising behaviour (Thabet *et al*. 2000). A minority of studies specifically aimed to evaluate the psychometric properties of the tools that they were using. This included a study in Kenya that translated and adapted the CBCL and then evaluated its validity and reliability prior to use (Kariuki *et al*. 2016) and a study in in Pakistan which specifically aimed to translate and validate the SDQ for children between 4 and 16-years-old (Samad *et al*. 2005). Some studies were more specifically using assessment tools to evaluate the impact of an intervention. A study in Turkey looked at the effect of zinc sulphate as an ADHD treatment and used the Du Paul Parents Rating of ADHD, the Attention Deficit Hyperactivity Disorder Scale and the Turkish Adaptation of Conners Teacher Questionnaire (Bilici *et al*. 2004; Rimal & Pokharel, 2016) to measure differences between the treated and non-treated groups.

### Types of tools identified

The Child Behaviour Checklist (CBCL) was the most commonly used tool in the studies found in our review, used in 26/82 (39%) of the studies included. The use of the CBCL was not limited to a determined region of the world, but it was the most popular tool across all regions (Table 3). It is of note, however, that the adaptation and validation of the CBCL was different in each setting with some studies describing exhaustive adaption and others not mentioning any changes at all. For example, a study in South Africa Sipsma *et al*. (2013), performed translations and back-translations, expert review and a pilot to validate a tool whereas a study in India exploring behaviour dysfunction in children used the CBCL with absolutely no details about its adaptation and validation for use in India (Prasad *et al*. 2014).

Some studies we found described the development of a new tool specifically for their population. The Child Behaviour Assessment Instrument (CBAI) is one example. It has been developed in Sri Lanka to screen young children at risk of behaviour problems (Samarakkody *et al*. 2010). This instrument was developed following a literature review and multiple reviews by expert panels and was found to be valid and reliable for its purpose (Cronbach's *α*  =  0.7). Similarly, Betancourt *et al*. (2010), developed and validated the Oxford Measure of Psychosocial Adjustment to be used to investigate the course of internalising and externalising behaviour problems amongst former child soldiers in Sierra Leone. A final example is the Independent Behaviour Assessment Scale (IBAS), which was constructed in Bangladesh through an ecological analysis of behaviours expected of children living in both rural and urban settings. The tool was then validated through a prior to ensure validity and reliability (Munir & Mcconuchie, 1999).

### Adaptation and validation of tools

Only one out of the 84 studies mentioned the use of the six stages recommended by the International Test Commission Guidelines for test translation and adaptation (Bartram & Muniz, 2016). Studies which do describe some type of adaptation procedure are highlighted in Table 4. At least half (46/84) of the studies made reference to adaptations or validation to justify the use of the tool in the setting. In some cases, a study mentioned the use of a validated tool for that setting but do not explain how it was validated (Emam, 2012; Matijasevich *et al*. 2014). Only ten of the 84 studies included in the review measured reliability in some way with most using a statistical measure such as Cronbach's *α* (to measure internal consistency) or test-retest reliability. Internal consistency will demonstrate how related the items are within the tool in its translated form when used to assess children in that setting (Al-Awad & Sonuga-Barke, 2002; Erdogan *et al*. 2008; Panter-Brick *et al*. 2009; Sánchez *et al*. 2011; Perera *et al*. 2012; Pires *et al*. 2012; Abdul Kadir *et al*. 2015; Shahini *et al*. 2015). Some studies evaluated the reliability of the tool to produce consistent results by measuring test-retest reliability (Al-Awad & Sonuga-Barke, 2002; Erdogan *et al*. 2008; Perera *et al*. 2012; Albores-Gallo *et al*. 2016). In addition, many researchers also explored the external validity and cross-cultural invariance that their findings had by comparing the prevalence of behavioural problems to those reported in similar settings.

**Table 4.** Analysis of the cultural adaption of the tools used through the Ecological Validity Framework

Author	Tool	Setting	Language	Persons	Metaphors	Content	Concepts	Goals	Methods	Context
Hartini *et al*. (2015)	CBCL	Indonesia	+	−	−	−	−	−	−	−
Albores-Gallo *et al*. (2016)	CBCL	Mexico	+	−	−	−	−	−	−	−
Bordin *et al*. (2013)	CBCL, YSR	Brazil	+	+	+	+	+	+	−	+
Eslami *et al*. (2010)	Adolescent Health and Development Questionnaire	Iran	+	−	−	−	−	−	−	−
Kariuki *et al*. (2016)	CBCL	Kenya								
Leung *et al*. (2006)	CBCL, TRF, YSR	China	+	−	−	−	−	−	−	−
Al-Awad & Sonuga-Barke (2002)	CTRS, CPRS	Sudan	+	+	−	−	+	−	−	−
Almaqrami & Shuwail (2004)	SDQ	Yemen	+	−	−	−	−	−	−	−
Bangirana *et al*. (2009)	CBCL	Uganda	+	−	−	−	−	−	+	−
Munir & McConachie, (1999)	Independent Behaviour Assessment Scale	Bangladesh	+	+	−	+	+	+	−	−
Ashenafi *et al*. (2001)	Diagnostic Interview of Children and Adolescent	Ethiopia	+	−	−	−	−	−	+	−
Erdogan *et al*. (1995)	CBCL	Turkey	+	−	−	−	−	−	−	−
Lambert & Lyubanksy (1999)	Jamaica Youth Checklist	Jamaica	+	+	+	+	−	−	−	−
Mpango *et al*. (2017)	Child and Adolescent Symptoms Inventory	Uganda	+	−	−	−	−	−	−	−
Menon *et al*. (2007)	SDQ	Zambia	+	−	−	−	−	−	−	−
Graham & Jorda, (2011)	SDQ	Vietnam	+	+	−	−	−	−	−	−
Dang *et al*. (2017)	CBCL	Vietnam	+	−	−	−	−	−	−	−
Omigbodun *et al*. (1996)	Child Behaviour Questionnaire	Nigeria	+	−	−	−	-	−	+	−
Omigbodun *et al*. (1996)	Reporting Questionnaire for Children	Nigeria	+	−	−	−	−	−	+	−
Samad *et al*. (2005)	SDQ	Pakistan	+	+	+	+	+	+	−	+
Mukherjee *et al*. (2014)	INDT – ADHD	India	+	+	+	+	+	+	−	+
Samarakkody *et al*. (2010)	Child Behaviour Assessment Instrument	Sri Lanka	+	+	+	+	+	−	−	+

**Table 5.** Table displaying the tools used to assess and screen for externalising behaviour problems in each country and for each age group

Assessment Tool	Countries used	Age of children
African Youth Psychosocial Assessment	Congo	7–18 years
BASC Behavioural Assessment System for Children	Guatemala	6–11 years
Behavioural Assessment System Children	Panama	6–11 years
BRIEF	Uganda	5–12 years
	India	3–7 years
CBCL	Egypt	6–12
	Mexico	1–5 years
	Uganda	5–12
	Ukraine	9–16
	India	<18 years, 3–18
	Vietnam	6–16 years
	Turkish	5–7 years, Preschool
	Romania	5–11 years
	Kenya	6–9 years
	Mauritius	
	South Africa	6–10, 7–11, 10–16 years
	Brazil	6–13 years, 6–16 years
	Thailand	3–5 years
	Kosovo	6–16 years
	Ethiopia	6–14 years
Child Global Assessment Scale (C-GAS)	Nigeria	15–18 years
Child Behaviour Assessment Instrument (CBAI)	Sri Lanka	4–6 years
CBQFP Child Behavior Questionnaire for Parents (CBQFP)	Kenya	6–9 years
	Nigeria	7–14 years
Child symptom inventory (SCI-4)	Iran	8–10 years
Child and Adolescent Symptom Inventory (CASI-5)	Uganda	
Connors		
Connor's Global Index – Child report	Jamaica	3–6 years
Turkish Conners (TACTQ)	Turkey	6–14 years
Conners Parent Symptom Questionnaire	China	6–12 years
Connor's Teacher rating scale	India	3–17 years, 6–15 years
Connor's	Egypt	Preschool
Conners' ADHD/Diagnostic and Statistical Manual for Mental DSM-		
IV Scales (CADS-T)	India	3–7 years
CTRS-39	Sudan	6–19 years
CPRS – 48	Sudan	6–19 years
Diagnostic Interview Schedule for Children	Ethiopia	5–14 years
	South Africa	7–11 years
DISYPS-KS (German ADHD rating scale)	Iran	6–14 years
DuPaul Parents Rating ADHD – Turkey version	Turkey	6–14 years
DSM-IV based parents' ADHD rating questionnaire	Iran	5–16 years
DSM Adapted Questionnaire	India	6–12 years
Eyberg Child Behavior Inventory (ECBI) – parent report	Jamaica	3–6 years
INCLEN Diagnostic Tool for Attention Deficit Hyperactivity Disorder (INDT-ADHD)	India	6–9 years
Independent Behaviour Assessment Scale (IBAS)	Bangladesh	2–9 years
Oxford Measure of Psychosocial Adjustment	Sierra Leone	10–17 years
Richman Child Behaviour Scale	South Africa	2 years, 2–4 years
Reporting Questionnaire for Children	Nigeria	7–14 years
Rutter Scale	Nigeria	7–14 years
SDQ	Malaysia	14–20
	Jamaica	3–6 years, 6 years
	Vietnam	6–16 years
	China	2–3 years
	Romania	5–11 years
	Russia	6–17 years
	Bangladesh	5 years
	South Africa	
	Zambia	11–15 years
	Afghanistan	5–16 years
	Nepal	11–17 years
	Pakistan	4–16 years, 5–12 years
	Gaza	3–16 years
	Palestine	6–15 years
Sutter-Eyberg Student Behavior Inventory (SESBI) – Teacher report	Jamaica	3–6 years
Swanson, Nolan and Pelham Questionnaire (SNAP-IV)	Brazil	6–16 years

We examined cultural adaptation of the tools used through the Ecological Validity Framework (Table 3). All versions of screening questionnaires used declared the use of appropriate language obtained through translation and back translation process and input from experts in the field. However, we found that efforts to consider the uniqueness of the culture to the content of the tool or adapts psychological concepts to societal constructs were not reported. Many authors claimed that they accounted for contextual features within the population they were studying comes from, although very rarely, did they disclose how they did consider this.

Those authors who developed new tools for use in their specific setting did fulfil the EVF and those that did, reported on their adjustment in language, metaphors, concepts and contents within their context. Two good examples of this were; the INCLEN Diagnostic Tool for Attention Deficit Hyperactivity Disorder (INDT-ADHD) (Mukherjee *et al*. 2014) and the CBAI in Sri Lanka (Samarakkody *et al*. 2010). In the former, the team ensured that translators ‘[*maintained*] *conceptual, content, semantic, operational and functional equivalence of the items*’ and in the latter, the team ensured that they defined behaviour problems through a literature search after ‘*considering the social, economic, historical and political context*’ of their setting.

## Discussion

Identifying children with behavioural problems, making diagnoses, supporting and managing the symptoms that these children have is incredibly important for the quality of life of children in LMIC. Without valid cross-cultural assessment and screening tools, clinicians may not appropriately diagnose children who need support. Similarly, researchers need to have valid and reliable tools to conduct robust and well-considered studies which provide better evidence both in epidemiological and intervention-based research for children with externalising behavioural problems in LMIC settings (Malhotra & Patra, 2014).

We found a large number of studies focusing on externalising behaviour in children and adolescents in LMIC. Despite this, researchers from LMIC settings still seem to favour tools for screening and assessment of externalising behaviours created in Western industrialised settings (Munir & Mcconuchie, 1999). This includes the SDQ screening tool and tools that assess the presence and severity of behaviours such as the CBCL and the Conners Rating Scales. Researchers may not yet know about the new tools have been created in LMIC settings and these tools may have not had much endorsement from other experts in the field. Furthermore, our review of the literature demonstrates how variable researchers can be in adapting or validating these tools for use in LMIC settings. We know that in many LMIC cultures, children with externalising disorders such as ADHD, are highly stigmatized particularly where obedience and respect for elders are often considered paramount (Abubakar *et al*. 2015). Identifying and supporting these children within a medical framework may be particularly helpful in some cases.

Our study demonstrates that there is a clear need for researchers working in LMIC settings to have tools which are both culturally appropriate and accurate (valid) for identifying the right kids with problems and furthermore, that are well-endorsed by others in the field as utilising good methods to demonstrate their validity. Although those working in global mental health recognise the importance of utilising tools with good cross-cultural validity, very few robustly validated tools are available for use around the world. Furthermore, the global mental health community has not yet endorsed or recommended any specific tools for use globally.

Our systematic review of the literature has shown that many researchers are using and have by using them, demonstrate their acceptance of some of these Western rating scales as being cross-culturally appropriate. The SDQ and CBCL have been widely used and translated into multiple languages. Although, researchers who have used tools such as the SDQ and CBCL claim to have ensured cross-cultural validity within their adapted (often just translated and back translated) versions, we would argue that the realities and day-to-day life of children in different settings might be quite different. Researchers who are using translated tools across settings may not always be identifying the same things in different places. For example, researchers using the same Arabic version of a tool in both Palestine and Kuwait may get non-comparable results as the life experiences of children living in those two countries may be quite different and therefore the way that parents answer the questions as understood, may vary. Another example is the use of the Spanish translation of the CBCL for a US-Spanish context, which may be a very different context to that in Central or South America (Rubio-Stipec *et al*. 1990; Ulloa *et al*. 2006). Although many LMIC are using Western standards to diagnose externalising behaviour using the DSM-V or ICD-10, often with the support of externalising behaviour rating scales or checklists (Liu, 2004), it is clear that more is needed to understand whether these tools are identifying children with similar features and constructs across countries. A number of studies have demonstrated that norms, beliefs, values and expectations of child behaviour in other parts of the world differ from that in Euro-American cultures (Levine & New, 2008; Kariuki *et al*. 2012; Mbuba *et al*. 2012; Lancy, 2015) and need to be taken into account when making diagnoses.

In some settings, this has been taken into account prior to conducting a study using a tool. For example, in Algeria (Petot *et al*. 2011) and Brazil (Saur & Loureiro, 2015) tools have been first adapted and then validated linguistically before further research was done. This process of adapting and validating an instrument for use in a new setting set a precedence in these settings for further research including that, which provided estimates of the true impact of externalising child behaviour problems in these populations. We would, therefore, advocate, not only for translation of tools but also for adequate processes that ensure the adaptation and contextualisation of the tool for the setting the child is living in. The International Test Commission (ITC) guidelines for Translating and Adapting tests is potential way of facilitating the adaption and contextualisation of tools to be used in a new setting (Bartram & Muniz, 2016). In addition, as societies continue to evolve it may be important to revise adaptation of tools to ensure that their applicability and validity continues (Almaqrami & Shuwail, 2004; Anwar, 2010).

### Methodology of tool adaptation

Tools may be good at identifying problems in child behaviour in one cultural setting but making sure they are right for the specific setting can have a big impact on the specificity and sensitivity of the tool. In most studies in our systematic review, we found that authors did not attempt adaptation or validation but merely translate the tool from English to the local language and then back-translated the tool to ensure translation was consistent and to ensure face validity. Many cross-cultural researchers have demonstrated how semantic equivalence may not necessarily be maintained if this is the limit of adaptation of a tool. This is particularly important when assessing mental health and behavioural problems as the diagnosis is so descriptive and dependent on conceptual understandings of those around the patient.

Very few researchers validated their tools to ensure the results obtained were correctly reflective of the construct being measured (externalising behaviour) in their setting (Table 3). Some researchers have assessed content validity through conducting a pilot study and consulting with local experts on how questions were interpreted (Pires *et al*. 2012). In one study, ‘expert help was provided to participants when completing the questionnaires to ensure all items were understood’ (Pal *et al*. 2011). Other researchers use cross-referencing to report on the internal and external consistency and reliability of tools by comparing their results on prevalence of externalising behaviours to that of Western populations. Many researchers solely justify their use of a tool based on a previous validation and focus purely on linguistic validity (Ashenafi *et al*. 2001; DiGirolamo *et al*. 2010; Walker *et al*. 2010; Kariuki *et al*. 2012). Furthermore, numerous authors have relied on the validation of tools that were conducted over a decade ago, not taking into account cultural changes that can occur with development and globalisation. We found that researchers were comfortable using tools validated by other groups without ensuring they would serve their purpose, instead of working on developing or adapting tools to be culturally appropriate. Despite this, many researchers have used the SDQ, CBCL and Conners rating scales widely in the field providing some face validity. However, this does not mean that they should not be adapted, piloted and validate to ensure they are measuring the right construct of child behaviour for each setting where researchers are studying child behaviour (Malhotra & Patra, 2014).

The International Test Commission has produced guidelines for Translating and Adapting Tests (2016) details how tool developers can translate and adapt new tools. We would recommend a staged process to translate, adapt and validate foreign tools, using this guideline as a framework, before introducing them in a new setting. This process should start by forward and back translating the content, then obtaining input from local experts in this area before running a pilot study putting this new tool into action. Following this adaptation process, a group of children with different characteristics should be selected to validate the tool. Furthermore, to ensure good validity, researchers should assess content and construct validity of any newly adapted tool. A six-step process for cross-cultural adaptation of self-report measures (Beaton *et al*. 2000) based on a review of adaptation procedures across medical, psychological and sociological literature is summarised in Appendix 1. Ultimately, we would welcome researchers using such processes to create or adapt tools for use in specific settings.

In our study, we aimed to examine systematically the cultural adaption of the tools using a framework to appraise against our findings. In doing so, we were dependent on what the authors reported in their papers. Although we know that culture can vary within a country particularly by ethnic group or socioeconomic status, we did not examine any high-income countries where low-income minorities may have been present. This would have been interesting but for this review, we focussed on LMICs, as this is where the mental health inequalities are greatest. For this review, available full texts published in both English and Spanish were included as one of the authors was a native speaker of both languages. We were, therefore, able to include studies from South America, where publishers had not translated articles into English.

Our review has identified a wide number of countries in LMIC settings, which are conducting research into externalising behaviour as well as a wide number of tools, which are used to screen and support diagnoses for these children (Table 2). We have evaluated the processes for adaption and validity of tools and rating scales used to assess externalising behaviour and have demonstrated some examples of good practice. We would advocate that more research be focussed on determining the reliability, validity and clinical effectiveness of adapted tools compared to tools that are specifically designed for Western target group. We would also promote researchers considering carefully how they could conduct studies to compare whether tools can work across countries to measure externalising behaviours in a similar way in order to compare outcomes in cross-country intervention studies. Alongside this, through reviews and consensus, we would encourage the experts in the global mental health community to provide more clarity as to which and what tools have robust measures of validity in cross-cultural use and to share this more widely through open access platforms for researchers in LMIC settings to utilise. A good example of this is the World Bank Toolkit, which provides a guide for researchers and programme officers as to the validity and use of measures of early childhood development in LMIC settings (Fernald *et al*. 2017).

## Key points

### What's known:

The rate of mental health problems in children in low and middle-income settings is high (Keiling *et al*. 2011). A substantial proportion of mental health difficulties (globally) in adults originate early in life, particularly externalising behaviour problems.

### What's new:

A wide number of LMIC countries are researching externalising behaviour problems in children. Common behavioural screening tools, checklists and rating scales are used in many settings but new and adapted measures of externalising behaviour have been validated in LMIC settings which may work across countries.

### What's clinically relevant:

Our study provides evidence that there is a need for culturally appropriate tools for screening and assessing behaviour in children in low income settings. It will be important for clinicians to check whether clear guidelines have been used in adapting, translating and validating tools (or items from tools) for use in their country or setting. The global mental health community may want to consider whether an open access platform providing information on the robustness and validity of different tools could enable clinicians to choose tools best suited to their setting.

## Supplementary material

For supplementary material accompanying this paper visit http://dx.doi.org/10.1017/gmh.2019.11. click here to view supplementary material
