# Supplementary material for: A systematic review of tools used to screen and assess for externalising behaviour symptoms in low and middle income settings
Source: Glob Ment Health (Camb). 2019 Jul 15;6:e13. doi: 10.1017/gmh.2019.11 (PMC6669966; doi:10.1017/gmh.2019.11)
Supplement: Supplementary file 1 [file S2054425119000116sup001.docx]

**Appendix 1:** Proposed framework for cross-cultural adaptations of questionnaires (adapted from Beaton et al., 2000; Bartram and Muniz, 2016)

Minimum two native English translators
Create 2 back translations

**Backward Translations**

New version of
tool developed

Cohort of participants complete questionnaire
Feedback on language and cultural meaning obtained
Construct and criterion validity
measured

Minimum two translators
1 knowledgeable on content of tool,
1 native in language and culture, and living in target locale

Resolve any discrepancies between translations

Made up of experts in the tool -
including local experts.
Make further changes to version

**Development of final version of tool**

**Stage 4**

**Stage 3**

**Pilot study**

**Forward Translations**

**Expert committee review tool**

**Synthesis new version of tool**

**Stage 2**

**Stage 1**
